# Targeting YAP in malignant pleural mesothelioma

**DOI:** 10.1111/jcmm.13182

**Published:** 2017-05-04

**Authors:** Wen‐Qian Zhang, Yu‐Yuan Dai, Ping‐Chih Hsu, Hui Wang, Li Cheng, Yi‐Lin Yang, Yu‐Cheng Wang, Zhi‐Dong Xu, Shu Liu, Geraldine Chan, Bin Hu, Hui Li, David M. Jablons, Liang You

**Affiliations:** ^1^ Thoracic Oncology Laboratory Department of Surgery, Comprehensive Cancer Center University of California San Francisco CA USA; ^2^ Department of Thoracic Surgery Beijing Chao‐Yang Hospital Affiliated with Capital University of Medical Science Beijing China; ^3^ Department of Thoracic Medicine Chang Gung Memorial Hospital Linkou, Taoyuan Taiwan; ^4^ Department of Respiration The Second Hospital of Shandong University Jinan China; ^5^ Department of Gastroenterology Shanghai General Hospital Shang Jiao Tong University Shanghai China

**Keywords:** mesothelioma, YAP, Hippo pathway, therapeutic target

## Abstract

Malignant mesothelioma is an aggressive cancer that is resistant to current therapy. The poor prognosis of mesothelioma has been associated with elevated Yes‐associated protein (YAP) activity. In this study, we evaluated the effect of targeting YAP in mesothelioma. First, we comprehensively studied YAP activity in five mesothelioma cell lines (211H, H2052, H290, MS‐1 and H2452) and one normal mesothelial cell line (LP9). We found decreased phospho‐YAP to YAP protein ratio and consistently increased GTIIC reporter activity in 211H, H2052 and H290 compared to LP9. The same three cell lines (IC_50_s < 1 μM) were more sensitive than LP9 (IC_50_ = 3.5 μM) to the YAP/TEAD inhibitor verteporfin. We also found that verteporfin significantly reduced YAP protein level, mRNA levels of YAP downstream genes and GTIIC reporter activity in the same three cell lines, indicating inhibition of YAP signaling by verteporfin. Verteporfin also impaired invasion and tumoursphere formation ability of H2052 and H290. To validate the effect of specific targeting YAP in mesothelioma cells, we down‐regulated YAP by siRNA. We found siYAP significantly decreased YAP transcriptional activity and impaired invasion and tumoursphere formation ability of H2052 and H290. Furthermore, forced overexpression of YAP rescued GTIIC reporter activity and cell viability after siYAP targeting 3′UTR of YAP. Finally, we found concurrent immunohistochemistry staining of ROCK2 and YAP (*P* < 0.05). Inhibition of ROCK2 decreased GTIIC reporter activity in H2052 and 211H suggesting that Rho/ROCK signaling also contributed to YAP activation in mesothelioma cells. Our results indicate that YAP may be a potential therapeutic target in mesothelioma.

## Introduction

Malignant pleural mesothelioma (MPM) is a very aggressive form of cancer that affects the pleura. It is mainly associated with occupational exposure to asbestos 1, but a genetic predisposition has also been implicated 2. In the United States, 70,000 new cases of mesothelioma are expected over the next 20 years 3. Most patients are diagnosed at a very advanced stage. Despite aggressive treatment with pleuropneumonectomy, chemotherapy and radiation therapy for eligible patients, the prognosis of mesothelioma is very poor, with a median survival of 11 months 4. Therefore, there is an urgent need to identify a feasible and effective therapeutic target for this fatal disease.

The Hippo (also known as the Salvador‐Warts‐Hippo) tumour suppressor pathway is a crucial regulator of organ growth, tissue regeneration and cell proliferation, and dysregulation of this pathway leads to tissue overgrowth and tumourigenesis 5. The transcriptional coactivator YAP is a major downstream negative effector of the Hippo pathway 6, 7 and is tightly negatively regulated by a series of upstream components such as NF2, LATS1/2, MST1/2 and SAV1, which are tumour suppressors in several types of human cancers 8. Recent studies suggest that the elevated YAP activity due to genetic inactivation of the Hippo pathway components may be associated with poor prognosis of patients with mesothelioma 9. The NF2 gene, which encodes the Merlin tumour suppressor protein, shows the highest frequency of inactivating mutation in the Hippo pathway of mesothelioma 10—nearly 50% of mesothelioma tumours according to one study 11. In addition, genetic inactivation status of LATS2, SAV1 and RASSF has been identified in several mesothelioma cell lines and clinical samples 12, 13. Deletion of these molecules caused by genetic inactivation leads to constitutive YAP activation and induces the oncogenic transformation in cooperation with distinct transcription factors such as TEAD family members (TEAD1‐4) 14, 15, 16, 17. More recently, studies of YAP modulation by the LIM‐domain protein AJUBA, a binding partner of LATS2, found that mesothelioma cells frequently showed loss of AJUBA expression, which contributes to aberrant YAP activation 18. Dysfunction of the Hippo pathway caused by such complicated factors eventually leads to dramatic increases in cell proliferation and metastasis that are largely dependent on YAP in mesothelioma. YAP activation status was identified in more than half of mesothelioma clinical samples in a 2011 study 19. Recently YAP constitutive activation was reported in 59% of MPM cohort 1 and in 24% of MPM cohort 2 patients, respectively 20.

The evidence thus far shows that the Hippo pathway and YAP are important in mesothelioma. Accordingly, we believe YAP may be a potential therapeutic target of mesothelioma. Therefore, in this study, we comprehensively evaluated the treatment effects of targeting YAP in mesothelioma cells.

## Materials and methods

### Cell lines and cell culture

Human mesothelioma cell lines 211H, H2052 and H2452 were purchased from American Type Culture Collections (ATCC, Manassas, VA, USA). Human mesothelioma cell lines H290 and MS‐1 were purchased from NIH (Frederick, MD, USA). Human normal mesothelial cell line LP9 was purchased from the Cell Culture Core Facility at Harvard University (Boston, MA, USA). All mesothelioma cell lines were maintained in RPMI‐1640 medium supplemented with 10% (v/v) heat‐inactivated foetal bovine serum (FBS) and penicillin (100 IU/ml). LP9 was maintained in M199 supplemented with 15% (v/v) heat‐inactivated FBS, 10 ng/ml EGF, 0.4 μg/ml hydrocortisone and penicillin (100 IU/ml). All cell lines were cultured at 37°C in a humid incubator with 5% CO_2_.

### Reagents

Antibody for YAP and phospho‐YAP (Ser127) used in this study were purchased from Cell Signaling, Inc. (Danvers, MA, USA). The SMARTpool siRNA targeting YAP (YAP siRNA‐1) was purchased from Thermo Scientific Dharmacon (Pittsburgh, PA, USA). Non‐targeting siRNA was used as control (Thermo Scientific Dharmacon). Another YAP siRNA (AM16708) targeting the 3′UTR end of the YAP gene (YAP siRNA‐2) was purchased from Life Technologies (Grand Island, NY, USA). The pcDNA4/HisMaxB‐YAP plasmid DNA used to overexpress the YAP gene in the cells was purchased from Addgene (Cambridge, MA, USA). The 8× GTIIC‐luciferase plasmid was purchased from Addgene and Renilla luciferase pRL‐TK plasmid was purchased from Promega (Madison, WI, USA). Verteporfin was purchased from Sigma‐Aldrich (St. Louis, MO, USA).

### Tissue samples and immunohistochemistry

Fresh mesothelioma and adjacent normal pleural tissues were obtained from patients with mesothelioma who were undergoing surgical resection of the primary tumour. Primary human mesothelioma samples from 60 patients were fixed in formalin and embedded in paraffin in 4‐μm tissue microarray sections. In seven of these patients, a small amount of normal pleura tissue had been obtained simultaneously to serve as control. All human tissue samples were obtained and analysed in accordance with procedures approved by the institutional review board of the University of California, San Francisco (IRB H8714–22 942–01). The sections were immunostained as previously described [21, 22]. The following scoring system was used: −, no stain; +, weak staining (≥10% and <30% stained cellularity considered as positive); ++, moderate staining (≥30% and <50% stained cellularity considered as positive); +++, strong staining (50% or above stained cellularity considered as positive). All scoring was carried out under a low power objective lens (20×) with a Zeiss Axioscop 2 microscope (Carl Zeiss Inc, Oberkochen, Germany) by two independent, blinded researchers. Images were taken under 10× or 20× objective lens.

### YAP siRNA transfection and verteporfin treatment

The mesothelioma cells were plated in 24‐well plates (for reporter assay) or 6‐well plates (for Western blot, PCR or wound‐healing assay) 24 hrs before treatment. Cells were transfected with 100 nmol/l of two siRNAs against YAP and control siRNA using Lipofectamine RNAiMAX (Invitrogen, Carlsbad, CA, USA) according to the manufacturer's protocol. After transfection for 48 hrs, cells were harvested for further analysis. Verteporfin was dissolved in DMSO. Cells treated with verteporfin (1 or 3 μM) or DMSO (0.1%) as a control were grown for 24 hrs before being harvested for further assays.

### Western blot analysis

Total protein was extracted from cell lines using M‐PER Mammalian Protein Extraction Reagent (Thermo Fisher Scientific, Waltham, MA, USA) supplied with Protease Inhibitor Cocktail Tablets (Roche, Lewes, UK), according to manufacturers’ protocols. The protein concentrations were measured with the Pierce BCA Protein Assay Kit (Thermo Fisher Scientific). A total of 20 μg of proteins were run on 4–20% gradient SDS‐polyacrylamide gels (Bio‐Rad Laboratories, Inc., Hercules, CA, USA) and transferred to Immobilon‐P nitrocellulose membranes (Millipore, Bellerica, MA, USA). The membranes were blocked in 5% non‐fat milk and then probed with the primary antibodies overnight at 4°C. The membranes were incubated with appropriate secondary antibodies, and then detected with an ECL blotting analysis system (Amersham Pharmacia Biotech, Piscataway, NJ, USA).

### Luciferase reporter assay

The 8× GTIIC‐luciferase plasmid (Addgene, Cambridge, MA, USA) and Renilla luciferase pRL‐TK plasmid (Promega) were cotransfected into cell lines. The transfection reagent was Lipofectamine 2000 (Invitrogen, Carlsbad, CA, USA). After 48 hrs, cells were lysed and the lysate was transferred into a 96‐well plate for analysis using the Dual‐Luciferase Reporter Assay Kit (Promega, Madison, WI, USA). Luminescent signaling was measured on a GloMax‐96 Microplate Luminometer (Promega) according to the manufacturer's instructions.

### RNA isolation, cDNA synthesis and quantitative real‐time RT‐PCR

Total RNA was extracted from cells using the RNeasy Mini kit (Qiagen, Valencia, CA, USA). The cDNA was transcribed from 500 ng of total RNA using iScript cDNA Synthesis Kits (Bio‐Rad), according to the manufacturer's protocol. The cDNA was used as the template for real‐time PCR detection using TaqMan Technology on an Applied Biosystems 7900HT sequence detection system (Applied Biosystems, Foster City, CA, USA). Expression of target genes and endogenous control gene b‐glucuronidase (*GUSB*) were detected using the probes commercially available (Applied Biosystems) and analysed using Relative Quantification Software SDS 2.4 (Applied Biosystems, Foster City, CA, USA).

### Cell viability assay

Cells were cultured in a 96‐well plate and treated with different doses of verteporfin or GSK269962A (0, 0.003, 0.01, 0.03, 0.1, 0.3, 1, 3, 10, 30, 100 μM). After 72 hrs of incubation, cells were lysed and luminescent signaling was generated by a CellTiter‐Glo Luminescent Cell Viability Assay reagent (Promega). Luminescent signaling was measured on the GloMax‐96 Microplate Luminometer. Proportional cell viability was analysed with GraphPad Prism6 software (GraphPad Software, Inc., La Jolla, CA, USA), which was used to calculate dose‐response curves and IC_50_.

### Transwell invasion assay

The transwell invasion assay was performed in a 6‐well plate transwell system (Corning Incorporated, Corning, NY, USA). The transwell inserts were coated with 300 μl matrigel and incubated at 37°C for half an hour. H2052 and H290 cells were harvested and 5 × 10^5^ cells were resuspended in serum‐free medium supplemented with verteporfin (1 or 3 μM) or DMSO (0.1%) to the upper chamber of the transwell. H2052 and H290 cells treated with control siRNA or YAP siRNA were harvested and 5 × 10^5^ cells resuspended in serum‐free medium. The lower chamber was infused with 2.6 ml complete growth medium (10% FBS). The transwell was incubated at 37°C for 20 hrs, at which point the gel and cells in the upper chamber were wiped out. After formalin fixation and methanol permeabilization, the insert membrane was stained by Crystal Violet (Sigma‐Aldrich) for 20 min. Phase contrast images were taken and the cells on the lower side of the membrane were counted in six random visual fields under a 20× objective lens.

### Tumoursphere assay

One cell per μl H290 single‐cell suspensions after treatment with control siRNA or YAP siRNA‐1 for 48 hrs or after treatment with 0.3 μM verteporfin, 1.0 μM verteporfin or DMSO (0.1%) for 24 hrs were prepared in StemPro MSC SFM Basal Medium CTS + StemPro MSC SFM Supplement CTS (Life Technologies), 2 nM L‐glutamine and penicillin (100 IU/ml). Then, 200 μl (200 cells per well) of the cells were plated in 96‐well ultra‐low attachment plates (Corning Incorporated). For each treatment, seed cells into the wells of two rows for a total of 20 wells. Tumoursphere were cultured for 7 days. Tumoursphere formed in non‐adherent cultures were counted under a 10× objective lens. The cut‐off size for the spheres counted was 60 μm.

### Statistical analysis

Data are presented as mean ± standard deviation (S.D.) from three independent experiments. All statistical analyses were performed using the GraphPad Prism (Version 5.0; GraphPad Software, San Diego, CA, USA). Student's *t*‐test was used for comparison between two groups. One‐way anova followed by Scheffe multiple comparisons were used to compare the differences among multiple groups. Chi‐square test was used for correlation analysis between the expression of YAP and ROCK1, YAP and ROCK2, ROCK1 and ROCK2. A significant difference was considered when the *P* value from a two‐tailed test was <0.05.

## Results

### YAP activity, GTIIC reporter activity and sensitivity to the YAP inhibitor verteporfin increased in several mesothelioma cell lines

**Figure 1:**
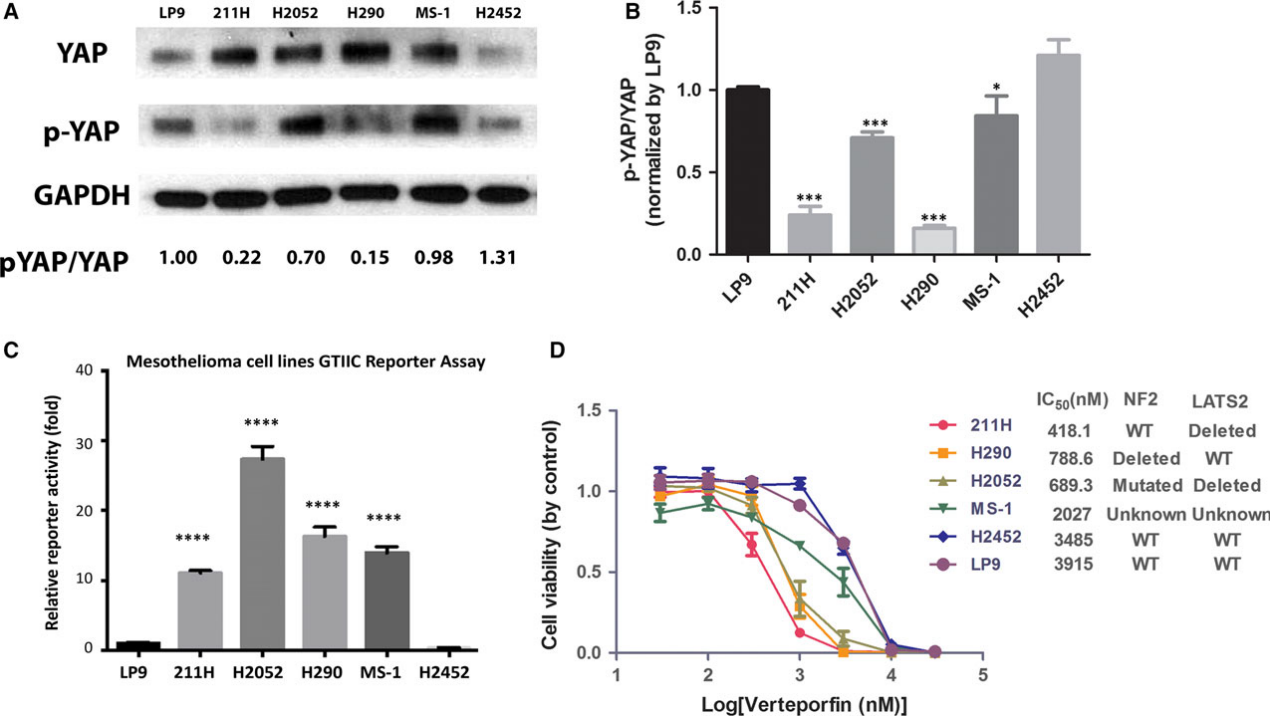
YAP activation and GTIIC reporter activity in cell lines. Five mesothelioma cell lines (211H, H2052, H2452, H290, MS‐1) and one normal mesothelial cell line (LP9) were measured by Western blotting and GTIIC reporter assay. (**A**) Western blot analysis of YAP and phospho‐YAP Ser127 (p‐YAP) in cell lines. (**B**) p‐YAP/YAP ratios were measured and are shown. Lower p‐YAP/YAP ratio indicates higher YAP activation status (* *P* < 0.05, *** *P* < 0.001, One‐way ANOVA and Scheffe multiple comparisons). (**C**) GTIIC reporter activity of the Hippo pathway in cell lines (**** *P* < 0.0001, One‐way anova and Scheffe multiple comparisons). (**D**) Cell viability analysis in 211H, H2052, H290, MS‐1, H2452 and LP9 cell lines after verteporfin treatment. IC_50_ and genetic inactivation status 13 are shown.

To investigate YAP activation in mesothelioma cell lines, we analysed the phosphorylation status phospho‐YAP (Ser127) and total amount of YAP in 211H, H2052, H2452, H290 and MS‐1 cells and in a normal mesothelial cell line, LP9, by Western blotting. We found that the YAP phosphorylation level to total YAP level ratio was significantly reduced in three mesothelioma cell lines (211H, H2052 and H290) compared with LP9 (Fig. 1A and B). To directly measure the activity of YAP‐TEAD‐mediated transcription, we used a GTIIC‐luciferase reported construct 23, which carries eight copies of the minimal TEAD‐binding sequences. We found that GTIIC reporter activity was remarkably elevated in the same three cell lines plus MS‐1, relative to LP9 (Fig. 1C), which suggests that these four mesothelioma cell lines have aberrantly high transcriptional activity of the Hippo pathway and YAP activation status compared with normal cell line LP9. We next tested the effects of verteporfin treatment on the cell viability of mesothelioma cells by treating 211H, H2052, H290, MS‐1, H2452 and LP9 cell lines with different doses for 72 hrs. Cell viability was assayed and IC_50_ of each cell line was calculated based on the dose‐response curves (Fig. 1D). These results show that verteporfin treatment suppressed cell viability in a dose‐dependent manner. Of the five cell lines tested, three showed high sensitivity to verteporfin treatment: the IC_50_ of verteporfin was 418.1 nM in 211H cells, 689.3 nM in H2052 cells and 788.6 nM in H290 cells. In contrast, the IC_50_ of verteporfin was much higher in MS‐1 (2027 nM), H2452 cells (3485 nM) and LP9 (3915 nM). The genetic inactivation status of Hippo pathway was referred to the previous publications 11, 12, 13 and was listed on Figure 1D.

### Verteporfin down‐regulates YAP protein expression, GTIIC reporter activity and downstream gene transcription of mesothelioma cells

**Figure 2:**
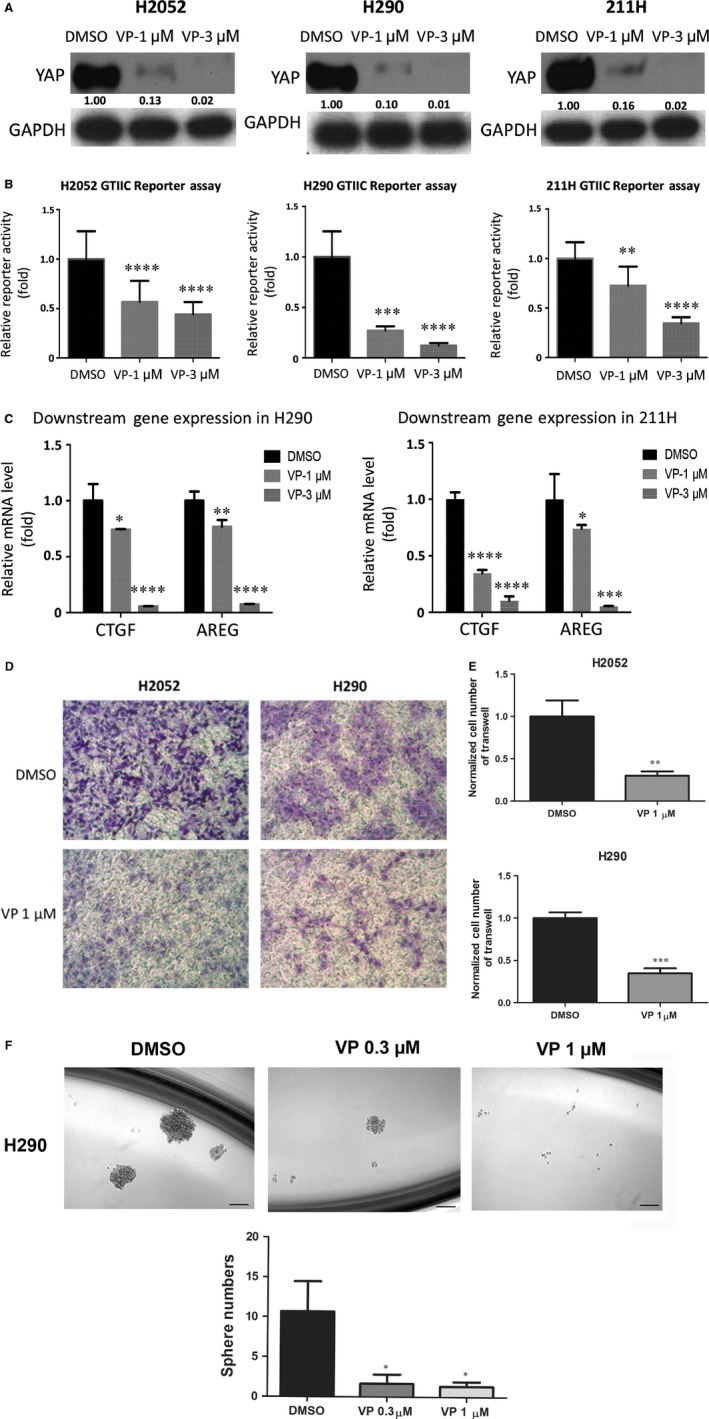
Analysis of YAP protein level, GTIIC reporter activity, mRNA level of CTGF, cell invasion and tumoursphere formation in mesothelioma cells. (**A**) Western blotting analysis of YAP expression in H2052, H290 and 211H cells treated with the YAP inhibitor verteporfin. (**B**) A dose‐dependent decrease in GTIIC reporter activity of the Hippo pathway was analysed in H2052, H290 and 211H cells (**P < 0.01, ****P < 0.0001, One‐way anova, Scheffe multiple comparisons). (**C**) Decreased mRNA levels of CTGF and AREG, the downstream genes of the Hippo pathway, in H2052, H290 and 211H cells (*P < 0.05, **P < 0.01, ****P < 0.0001, One‐way anova, Scheffe multiple comparisons). (**D**) Decrease in cell invasive ability after verteporfin treatment in H2052 cells. Images were taken under a 20 × objective lens. (**E**) Quantitative analysis of the number of cells that invaded the lower side of the membrane in each experimental group (**P < 0.01, ***P < 0.001, Student's *t*‐test). (**F**) Decrease in sphere formation ability in H290 cells after verteporfin treatment. Images were taken under a 10 × objective lens. (**G**) Quantitative analysis of tumoursphere assay shows verteporfin treatment decreased tumoursphere formation ability in H290 cells (*P < 0.05, One‐way anova and Scheffe multiple comparisons).

We analysed YAP protein level in mesothelioma cells H2052, H290 and 211H cell lines after treatment with the current commercially available YAP inhibitor verteporfin. We chose H2052, H290 and 211H for further analysis due to their known genetic mutations of Hippo pathway components, and high YAP and reporter activity level. The results showed that YAP protein level clearly decreased in a dose‐dependent manner in these three cell lines treated with verteporfin for 24 hrs, in contrast to control treatment with DMSO (Fig. 2A).

We then examined the effect of verteporfin on GTIIC reporter activity. After treatment with verteporfin, GTIIC reporter activity decreased in a dose‐dependent manner in H2052, H290 and 211H cells, as compared to the DMSO control (Fig. 2B, *P* < 0.0001). Semiquantitative RT‐PCR analysis also showed a dose‐dependent decrease of CTGF and AREG transcription in H290 and 211H cell lines (Fig. 2C, *P* < 0.001). Together, these results suggest that verteporfin treatment reduces YAP protein level, down‐regulates reporter activity and downstream gene transcription of the Hippo pathway in mesothelioma cells.

To investigate the cause of YAP suppression by verteporfin, we analysed both YAP mRNA level and protein level change (Fig. S1). The results showed that 1 μM verteporfin treatment significantly reduced YAP mRNA level in H2052 and H290 cells but did not affect YAP mRNA level in 211H cells, indicating that verteporfin could suppress YAP at transcription level in certain cell lines. To examine whether degradation also contributed to YAP suppression by verteporfin, we treated the cells with proteasome inhibitor MG132 together with verteporfin and measured the change in protein level. As shown in Figure S1A, when proteasome degradation was inhibited by MG132, YAP protein level was increased compared to that for verteporfin treatment alone at 8 and 24 hrs time‐points in all three cell lines. This indicated verteporfin could suppress YAP through proteasome degradation.

### Verteporfin suppresses invasion and tumoursphere formation of mesothelioma cell H290 and H2052

To assess the effect of verteporfin on the invasive ability of mesothelioma cells, we carried out a transwell assay using H2052 and H290 cells. We did not include 211H in these assays due to its high sensitivity to verteporfin. 1 μM verteporfin treatment to 211H for 20 hrs killed over 50% of 211H cells, which complicated the interpretation of the transwell and sphere formation results. We found that 1 μM verteporfin for 20 hrs significantly reduced the number of cells that migrated to the bottom of transwell membranes, indicating that verteporfin inhibited the invasive ability of H2052 and H290 (Fig. 2D and E). 0.3 μM verteporfin for 24 hrs treatment did not significant reduced cell viability of H2052 and H290. 1 μM verteporfin for 24 hrs treatment reduced cell viability of H2052 and H290 to 85.4% and 88.3% of control, respectively (Fig.S2A and B).

To measure the effect of verteporfin on the self‐renewal of cancer stem cells in mesothelioma, we used a tumoursphere assay. Under our experimental conditions, H2052 cells could not form compact spheres after 1 week of incubation. H290 tumoursphere were treated with verteporfin (0.3, 1 μM), and the effect on cancer stem cells was determined. Tumoursphere formation efficiency decreased significantly and in a dose‐dependent manner after verteporfin treatment (Fig. 2F and G).

### YAP inhibition by siRNAs down‐regulates GTIIC reporter activity of mesothelioma cells and mRNA level of YAP downstream genes

**Figure 3:**
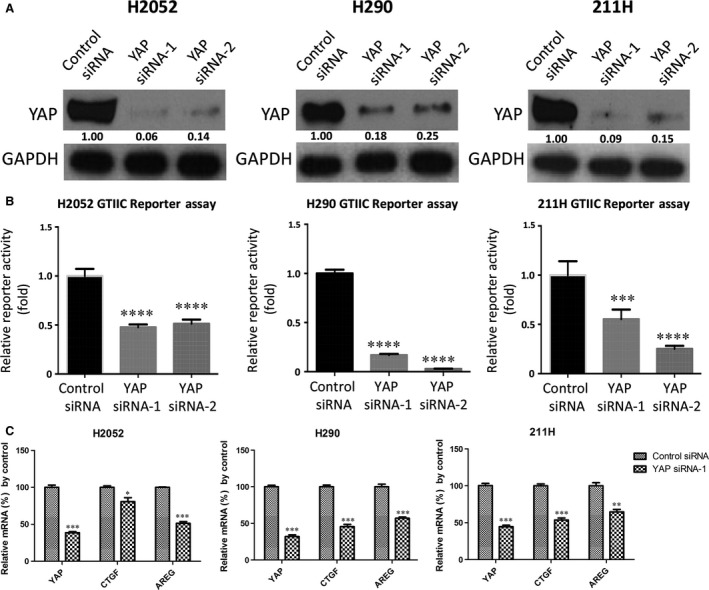
Analysis of YAP protein level, GTIIC reporter activity and mRNA level of YAP and its downstream genes after YAP inhibition by siRNAs. (**A**) Decreased YAP protein level after YAP siRNA‐1 and YAP siRNA‐2 treatment in H2052, H290 and 211H cells. (**B**) GTIIC reporter activity of the Hippo pathway after YAP inhibition by the two siRNAs was analysed in H2052, H290 and 211H cells (***P < 0.001, ****P < 0.0001, one‐way anova and Scheffe multiple comparisons). (**C**) Decreased mRNA levels of CTGF and AREG, the downstream genes of the Hippo pathway, in H2052, H290 and 211H cells treated with the YAP siRNA‐1 (*P < 0.05, **P < 0.01, ***P < 0.001, Student's *t*‐test).

As YAP is a central effector of the Hippo pathway, we next investigated whether direct YAP suppression affects the reporter activity of this pathway in mesothelioma cells. First, we examined the protein level of YAP by assessing the efficiency of YAP inhibition by two types of siRNAs (YAP siRNA‐1 and YAP siRNA‐2) after 48 hrs in H2052, H290 and 211H cells by Western blotting. YAP expression clearly decreased in these siRNA‐treated cell lines, in contrast to what occurred after control treatment (Fig. 3A). Second, analysis of GTIIC reporter activity of the Hippo pathway showed that YAP inhibition by siRNA significantly decreased GTIIC reporter activity of the Hippo pathway in these three cell lines (*P* < 0.0001) compared to that of their respective control cells treated with non‐targeting siRNA (Fig. 3B). Third, analysis of mRNA level of YAP downstream gene after knockdown of YAP with siRNA indicated YAP deprivation significantly reduced the mRNA level of YAP downstream gene CTGF and AREG (Fig. 3C).

### Knockdown of YAP with YAP siRNA impaired invasion and tumoursphere formation ability of H290 and H2052 mesothelioma cell lines

**Figure 4:**
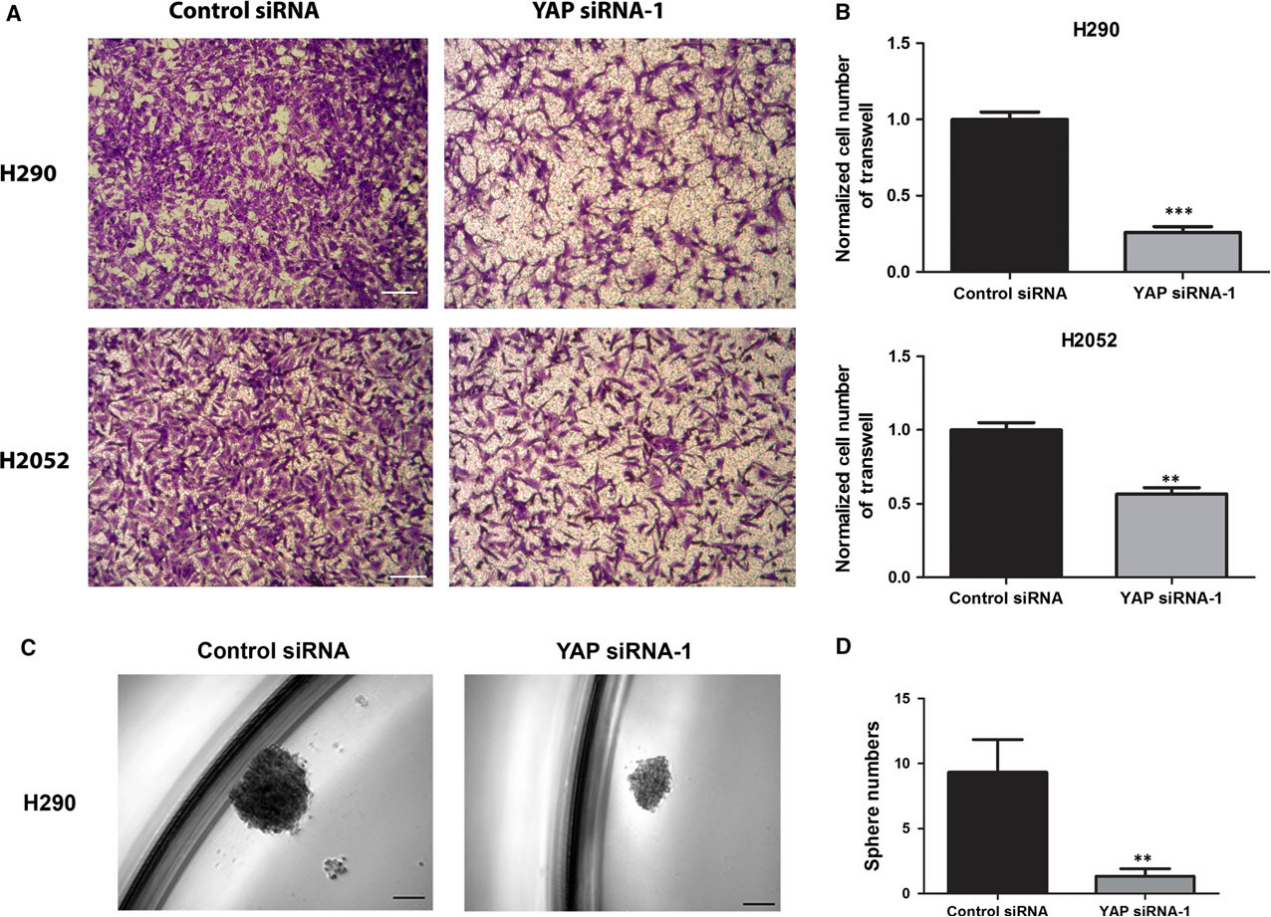
Analysis of invasion and tumoursphere formation after YAP siRNA‐1 treatment in mesothelioma cells. (**A**) Decrease in cell invasion ability in H2052 and H290 cells after YAP siRNA‐1 treatment. Images were taken under a 20 × objective lens. (**B**) Quantitative analysis of the number of cells that invaded the lower side of the membrane in each experimental group (**P < 0.01, ***P < 0.001, Student's *t*‐test). (**C**) Decrease in sphere formation ability in H290 cells after YAP siRNA‐1 treatment. Images were taken under a 10 × objective lens. (**D**) Quantitative analysis of tumoursphere assay shows verteporfin treatment decreased tumoursphere formation ability in H290 cells (**P < 0.01, Student's *t*‐test).

The same number of H290 and H2052 cells treated with control or YAP siRNAs for 48 hrs were cultured for the invasion and tumoursphere formation assay. The invasion assay indicated that knockdown of YAP significantly reduced the invasion ability of H90 and H2052, respectively to 26.2% and 56.7% compared to cells treated with control siRNA (Fig. 4A and B). The YAP siRNA‐1 treatment did not significantly reduce cell viability (Fig. S2C and D). For the tumoursphere assay, under our experimental conditions, H2052 cells could not form compact spheres after 1 week incubation. Currently, we do not know the reason for this. However, H290 cells nicely formed compact spheres. Around 10 spheres larger than 60 μm formed when 4000 H290 cells were treated with Control siRNA after 1 week incubation. When H290 cells were treated with YAP siRNA, the sphere number significantly reduced to 2 (Fig. 4C and D). The YAP siRNA‐1 treatment did not reduce cell viability significantly (Fig. S2C). The invasion and tumoursphere formation data indicated that YAP deprivation significantly impaired the invasion ability and the self‐renewal ability of cancer stem cells of mesothelioma cell H290.

### Forced overexpression of YAP rescues GTIIC reporter activity and cell viability during YAP inhibition in mesothelioma cells

**Figure 5:**
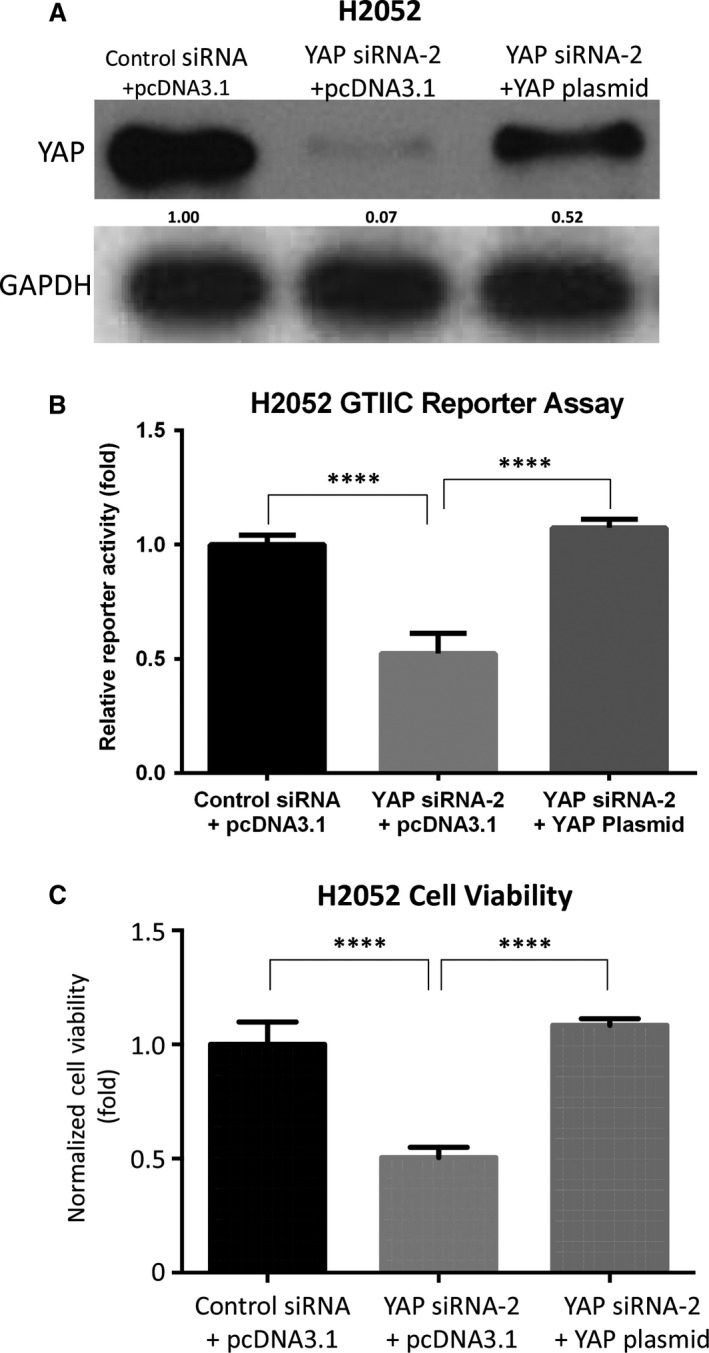
Forced overexpression of the YAP gene rescues GTIIC reporter activity and cell viability during YAP inhibition in H2052 cells. (**A**) Western blotting analysis of YAP and GAPDH after silencing by YAP siRNA‐2 (which targets the 3′UTR end of the YAP gene) and/or forced overexpression of the YAP gene in H2052 cells. (**B**) GTIIC reporter activity of the Hippo pathway after YAP silencing by YAP siRNA‐2 and/or forced overexpression YAP gene in H2052 cells (***P < 0.001, ****P < 0.0001, Student's *t*‐test). (**C**) Cell viability analysis after YAP silencing by YAP siRNA‐2 and/or forced overexpression of the YAP gene in H2052 cells (****P < 0.0001, Student's *t*‐test).

To further understand YAP involvement in the transcriptional activity of the Hippo pathway and cell viability of mesothelioma, we analysed GTIIC reporter activity and cell viability after YAP inhibition with or without forced overexpression of the YAP gene in H2052 cells. After forced overexpression of the YAP gene, YAP protein level was higher than in the cells treated with YAP siRNA‐2 (which targets the 3′UTR end of the YAP gene; Fig. 5A). After YAP siRNA‐2 treatment, GTIIC reporter activity and cell viability were significantly reduced by 47.7% and 49.7%, respectively, compared to cells treated with control non‐targeting siRNA (*P* < 0.0001), and GTIIC reporter activity and cell viability were rescued by 67.4% and 58.1%, respectively after forced overexpression of the YAP gene (*P* < 0.0001; Fig. 5B and C). Together, these results suggest that GTIIC reporter activity and cell viability can be positively regulated by YAP expression in mesothelioma cells.

### Concurrent overexpression of YAP and ROCK2 in mesothelioma tumours

**Figure 6:**
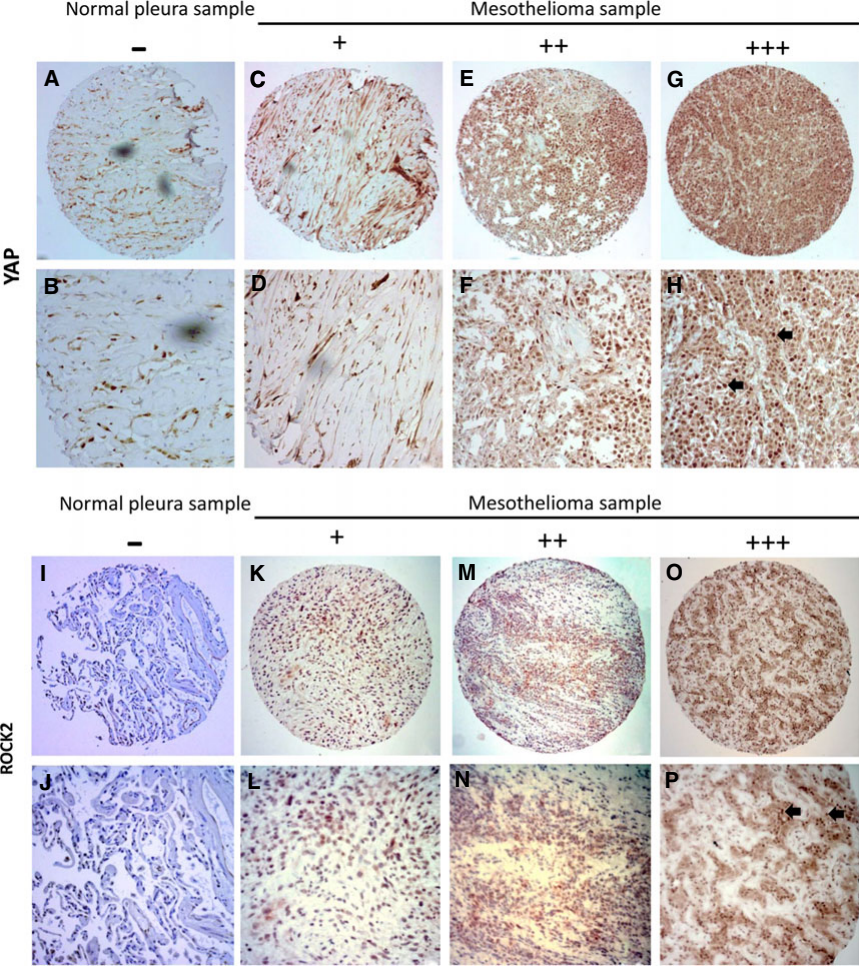
Immunohistochemistry of YAP and ROCK2 staining in mesothelioma and normal pleura samples. (**A, B**) Normal pleura sample. (**C‐H**) Mesothelioma samples. (**C, D**) Weak stain. (**E, F**) Moderate stain. (**G, H**) Strong stain. Staining of YAP was localized in nuclei (arrows) of mesothelioma under a 20 × objective lens. (Scale bar: 180 μm). (**I, J**) Normal pleura sample. (**K‐P**) Mesothelioma samples. (**K, L**) Weak stain. (**M, N**) Moderate stain. (**O, P**) Strong stain. (**P**) Staining of ROCK2 was localized in nuclei (arrows) of mesothelioma under 20 × objective lens. (Scale bar: 180 μm).

Rho GTPases have critical roles in regulating dynamics of the actin cytoskeleton and promoting cell proliferation. Previous studies have revealed that RhoA strongly enhances YAP/TAZ activity 23, 24. To gain insights into the mechanisms underlying elevated YAP activity in pleural mesothelioma, we examined YAP, ROCK1 and ROCK2 expression levels in mesothelioma tissue samples from 60 patients using immunohistochemistry. The positive and negative results of YAP, ROCK1 and ROCK2 staining are shown in Figure 6, Figure S3 and Table 1. Among the 60 mesothelioma samples analysed, YAP staining in nucleus was negative (−) in 6.7%, weak (+) in 25.0% and moderate to strong (++/+++) in 68.3% (Fig. 6A–H, Table 1). ROCK1 staining in the nucleus was negative (−) in 3.3%, weak (+) in 35.0% and moderate to strong (++/+++) in 61.7% (Fig. S3A–H, Table 1). ROCK2 staining in the nucleus was negative (−) in 5.0%, weak (+) in 15.0% and moderate to strong (++/+++) in 80.0% (Fig. 6I–P, Table 1). We found that ROCK2 staining was concurrent with strong nuclear staining of YAP (*P* < 0.05, chi‐square test; Table 2); ROCK2 was concurrent with strong nuclear staining of ROCK1 (*P* < 0.05, chi‐square test). However, we did not find significant concurrent expression of ROCK1 with YAP.

**Table 1:** Positive and negative number and ratio of YAP in 60 primary mesothelioma samples

	−Number(ratio)	+Number(ratio)	++Number(ratio)	+++Number(ratio)
YAP	4 (6.7%)	15 (25.0%)	25 (41.7%)	16 (26.7%)
ROCK1	2 (3.3%)	21 (35%)	30 (50%)	7 (11.7%)
ROCK2	3 (5%)	9 (15%)	37 (61.7%)	11 (18.3%)

**Table 2:** Chi‐square analysis of correlation between YAP, ROCK1 and ROCK2

	ROCK1(−/+)	ROCK1 (++/+++)
YAP (−/+)	8	11
YAP (++/+++)	4	37
		*N* = 60, *P* = 0.12

	ROCK2(−/+)	ROCK2 (++/+++)
YAP (−/+)	8	11
YAP (++/+++)	4	37
		*N* = 60, *P* < 0.05

	ROCK1(−/+)	ROCK1 (++/+++)
ROCK2 (−/+)	11	1
ROCK2 (++/+++)	12	36
		*N* = 60, *P* < 0.05

### Inhibition of RhoA/ROCK signaling suppresses GTIIC reporter activity and cell viability of mesothelioma cells

**Figure 7:**
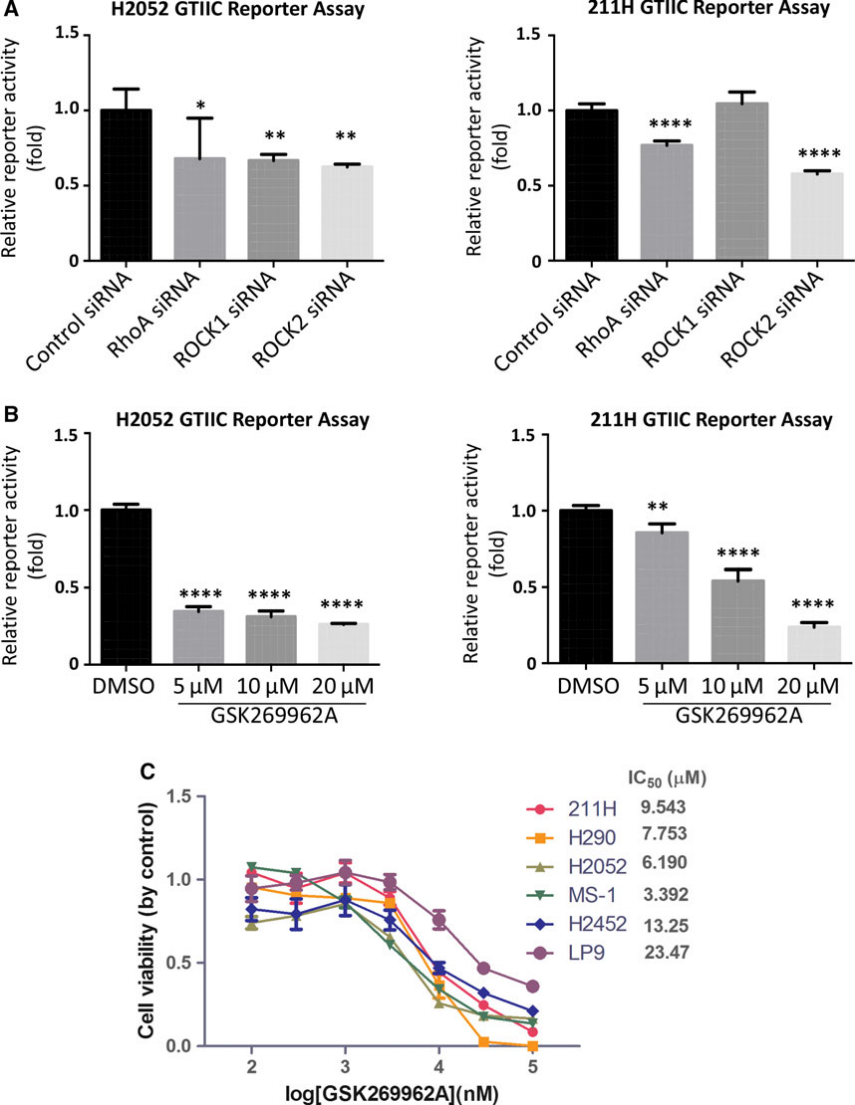
Inhibition of RhoA/ROCK signaling suppresses GTIIC reporter activity and cell viability of mesothelioma cells. (**A**) GTIIC reporter activity of the Hippo pathway after RhoA, ROCK1 and ROCK2 siRNA was analysed in H2052 and 211H cells (*P < 0.05, **P < 0.01, ****P < 0.0001, one‐way anova and Scheffe multiple comparisons). (**B**) GTIIC reporter activity of the Hippo pathway after different doses of ROCK inhibitor GSK269962A was analysed in H2052 and 211H cells (**P < 0.01, ***P < 0.001, ****P < 0.0001, one‐way anova and Scheffe multiple comparisons). (**C**) Cell viability analysis in 211H, H2052, H290, MS‐1, H2452 and LP9 cell lines, after GSK269962A treatment.

Knockdown of RhoA or ROCK2 with specific siRNAs significantly decreased GTIIC reporter activity in H2052 and 211H compared with control siRNA (Fig. 7A). ROCK1 siRNA also significantly down‐regulated GTIIC reported activity in H2052, but not in 211H. Additionally treating mesothelioma cells with GSK269962A, a selective inhibitor of Rho‐Kinase, largely decreased GTIIC reporter activity in both H2052 and 211H, and this suppression effect of GSK269962A on YAP activity was dose‐dependent (Fig. 7B). More importantly, GSK269962A treatment decreased cell viability of 211H, H290, H2052, MS‐1, H2452 and LP9 in a dose‐dependent manner as well (Fig. 7C). Collectively, these findings indicate that activation of RhoA/ROCK signaling contributes to YAP hyperactivity in pleural mesothelioma and inhibitors of RhoA/ROCK could be used to suppress YAP activity and thereby decrease cell viability of mesothelioma cells.

## Discussion

Nearly 75% of mesothelioma tumours contain genetic inactivation status of NF2 or downstream components of the Hippo pathway, which negatively regulates YAP activity 25, 26. This finding suggests that mesothelioma mostly relies on dysregulation of the Hippo pathway for tumour activity 27. The frequent mutations present in the Hippo pathway components indicate the feasibility of targeting this pathway for mesothelioma therapy. YAP is a candidate oncogene that has shown potential to be a therapeutic target 28, 29. We found that 68.3% of primary mesothelioma tumours showed aberrant YAP activation in nuclei (++/+++), a result consistent with results of previous studies 12, 13, 20. We did not find an association between YAP activation and overall survival or disease‐free survival of patients. The lack of association in our study may be because of the small sample size. A recent study found that YAP/TAZ immunoreactive score was correlated with overall survival of osteosarcoma patients 30. Among the five mesothelioma cell lines we studied, three (211H, H2052 and H290) showed both decreased phosphorylation of YAP (Ser127) to YAP ratio and increased GTIIC reporter activity. Importantly, all of these three cell lines reportedly contain genetic inactivation status of the Hippo pathway components: H290 with NF2 homozygous deletion, 211H with LATS2 deletion, and H2052 with both NF2 mutation and LAST2 deletion 19. The results of our study support that these mutations lead to aberrant YAP activation and elevated transcriptional activity of the Hippo pathway in several mesothelioma cell lines.

In our study, YAP inhibition by siRNAs significantly suppressed the transcriptional activity of the Hippo pathway in mesothelioma cells and this suppression could be rescued by forced overexpression of YAP in the cells. Another study 13 reported that specific targeting of YAP by siRNAs inhibits mesothelioma cell growth. Furthermore, we found that YAP inhibition by siRNA impaired the invasion and tumoursphere formation ability of mesothelioma cell H290 an H2052. These findings suggest that YAP, as the major mediator of the Hippo pathway 31, 32, is involved in the elevation of transcriptional activity, and in turn controls mesothelioma cell proliferation, invasion and stem cell renewal abilities. Our data indicate that YAP is a potential therapeutic target in mesothelioma and deprivation of YAP could suppress tumour progression in mesothelioma.

**Figure 8:**
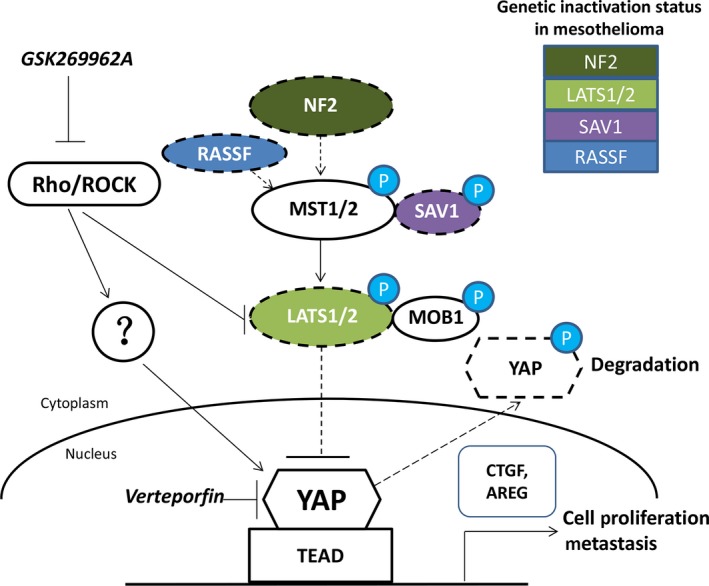
Diagram of the Hippo pathway in mesothelioma cells. Activated NF2 turns on Hippo kinase activity. With the genetic inactivation of Hippo kinases, YAP enters the cell nucleus instead of being phosphorylated, and promotes Hippo downstream gene transcription, cell proliferation and metastasis. Verteporfin disrupts the formation of the YAP–TEAD complex by specific binding to YAP and changing its conformation, thereby blocking the transcription of downstream genes (such as CTGF and AREG), then inhibiting the mesothelioma cell proliferation and metastasis. RhoA/ROCK signaling enhances YAP activity through inhibiting LATS1/2 and other undefined pathways. Rho/ROCK inhibitor GSK269962A prevents the activation of YAP by Rho/ROCK.

Verteporfin, a benzoporphyrin derivative, has been identified as a YAP inhibitor 33. Verteporfin is in clinical use as a photosensitizer in photodynamic therapy for neovascular macular degeneration as well as treatment for several human cancers, after it is activated by laser light to generate reactive oxygen radicals that eliminate abnormal blood vessels 34, 35. Verteporfin selectively binds YAP, thus altering YAP conformation and abrogating its interaction with TEAD, and in turn inhibits the oncogenic activity of YAP 36, 37. As a YAP inhibitor, verteporfin does not require light activation 38. Previous studies have noted that verteporfin alters YAP conformation, thus inducing the degradation of YAP protein by protease 33. The mechanism for YAP inhibition by verteporfin was through up‐regulating 14‐3‐3σ, sequestering YAP in the cytoplasm and leading to its degradation 39. We previously found that verteporfin can efficiently reduce YAP protein level in lung cancer cells 40. In the current study, we found that verteporfin treatment significantly reduced YAP protein in mesothelioma cells not only through proteasome degradation but also through transcriptional down‐regulation. To our knowledge, this is the first comprehensive evaluation of the effect of verteporfin treatment in mesothelioma cells. Our results show that 211H, H2052 and H290 cells are highly sensitive to verteporfin treatment. The IC_50_ of verteporfin is less than 1 μM in these three cell lines. All of these findings suggest that clinical trials of verteporfin may be warranted in mesothelioma patients. The high sensitivity of 211H, H2052 and H290 may be due to their aberrant YAP activation, elevated transcriptional activity and known mutations of the Hippo pathway components. In other words, the mesothelioma cells with aberrant YAP activation due to the Hippo pathway dysfunction were extremely sensitive to verteporfin treatment (Figs 1D and 8). This provides potential approaches to select patients likely to benefit from verteporfin by checking Hippo pathway components mutations contributing aberrant YAP activation.

Hippo downstream genes, such as CTGF and AREG, have been associated with the occurrence and development of human cancer 22, 41, 42, 43, 44. Our study shows that in H290 and 211H cells, the expression of these Hippo downstream genes can be reduced by YAP siRNA or verteporfin treatment. Moreover, verteporfin also down‐regulates the GTIIC reporter activity and the protein level of YAP. Our results suggest verteporfin has previously unrecognized inhibition effects on YAP transcriptional activity in mesothelioma cells. Furthermore, we found that the migration and invasion of mesothelioma cells were inhibited after verteporfin treatment. Taken together, these results provide evidence that specific YAP inhibition could suppress tumour progression in mesothelioma.

Cancer stem cells have been implicated in tumourigenesis, recurrence and metastasis, but effective therapeutic strategies that target these cells are lacking 45, 46, 47. The Hippo pathway is involved in cancer stem cell self‐renewal, differentiation and tumourigenesis 48. Sphere formation assay has been widely used to identify stem cells by evaluating the self‐renewal ability of a cell vitro. Our sphere formation assay result showed that YAP siRNA and verteporfin treatment inhibited tumoursphere formation in H290. This finding indicated that YAP inhibition may suppress tumour progression through the suppression of cancer stem cells in mesothelioma.

Two groups reported the off‐target effects of verteporfin on YAP 49, 50, 51. The off‐target effects of verteporfin require the further modification of verteporfin to increase specificity. Another option is to find potential druggable targets that can positively regulate YAP. The Hippo pathway comprises a large network of proteins and RhoA/ROCK signaling has been shown to enhance YAP activity by inhibiting LATS1/2 52, 53. Our study shows that strong staining of ROCK2 was concurrent with strong nuclear staining of YAP. Inhibition of RhoA/ROCK signaling by siRNAs or inhibitors decreased YAP transcriptional activity. Interestingly, in H2052, LATS2 is mutated and LATS1 is wild‐type; in 211H cells, both LATS1 and LATS2 are mutated. These mutations in LATS1/2 resulted in partial or total inactive regulation of YAP through LATS1/2 in these cells. However, YAP transcriptional activities of both cell lines were responsive to RhoA/ROCK signaling inhibition. Collectively, our results suggest that Rho/ROCK could regulate the Hippo pathway through additional mechanisms besides LATS1/2, and that kinase inhibitors of Rho/ROCK may have therapeutic roles for mesothelioma treatment through regulation of the Hippo pathway. Finally, our study suggests that ROCK2, but not ROCK1, may play a major role in regulating YAP. ROCK2 expression correlated significantly with YAP expression in MPM patients, but ROCK1 expression did not. ROCK2 siRNA inhibited GTIIC reporter activity in both H2052 and 211H cells, but ROCK1 siRNA only inhibited activity in H2052 cells.

Our proposed diagram of the regulation of YAP by the Hippo pathway and Rho/ROCK signaling in mesothelioma cells (Fig. 8) shows that the upstream tumour suppressor NF2 or RASSF can activate the Hippo core kinase cascade through phosphorylation of MST1/2 26, 44. Activated MST1/2 kinases associate with their partner SAV1 and phosphorylate and activate LAST1/2 and their partner MOB1. Then, activated LATS1/2 kinases phosphorylate and inhibit YAP by promoting its cytoplasmic retention and degradation. However, when the tumour suppressors NF2, LATS1/2, SAV1, RASSF are inactivated due to genetic mutation, fusion or deletion 13, YAP can translocate and accumulate in the nucleus and interact with transcription factor TEAD and initiate the gene transcription involved in anti‐apoptosis and proliferation, for example, that of CTGF and AREG. The small molecule verteporfin can disrupt the formation of the YAP‐TEAD complex and then block transcription of the YAP target gene. RhoA/ROCK signaling enhances YAP activity through inhibiting LATS1/2 kinases and other undefined pathways. Rho/ROCK inhibitor GSK269962A prevents the activation of YAP and inhibited transcriptional activity of YAP‐TEAD.

In summary, mesothelioma shows frequent YAP activation due to genetic inactivation of the Hippo pathway components. Specific targeting of YAP has inhibitory effects on human mesothelioma cells. Our findings suggest that YAP is an oncogenic driver and may be a potential therapeutic target in mesothelioma.

## Author Contributions

WQZ, YYD, DJ and LY conceived and designed the experiments. WQZ, YYD and GC carried out experiments. PCH, LC and YLY analysed the data. YLY, YCW, BH, LS and LH contributed materials tools. WQZ and YYD organized and wrote the manuscript. LY and ZDX reviewed and revised the manuscript. DJ gave important directions to the study and revised the manuscript. All authors had final approval of the submitted and published versions.

## Conflict of interest

The authors confirm that there are no conflicts of interest.

## Supporting information


**Fig. S1** Analysis of YAP protein level and mRNA level with 1 μM verteporfin treatment alone or 1 μM verteporfin with 20 μM MG132.

**Fig. S2** Cell viability of H2052 (**A**) and H290 (**B**) treated with verteporfin for 24 hrs. (*P < 0.05, ****P < 0.0001, one‐way anova and Scheffe multiple comparisons). H290 and H2052 cells were treated with control siRNA or YAP siRNA‐1 for 24 hrs. Equal cell numbers (5000 cells in 500 μl) of H290 and H2052 were seeded in 96‐well plates. After 20 hrs, cell viability of H290 (**C**) and H2052 (**D**) were analysed. After 7 days, cell viability of H290 (**F**) was analysed.

**Fig. S3** Immunohistochemistry of ROCK1 staining in mesothelioma and normal pleura samples.
