# Supplementary figures and images for: Targeting YAP in malignant pleural mesothelioma
Source: J Cell Mol Med. 2017 May 4;21(11):2663–76. doi: 10.1111/jcmm.13182 (PMC5661117; doi:10.1111/jcmm.13182)

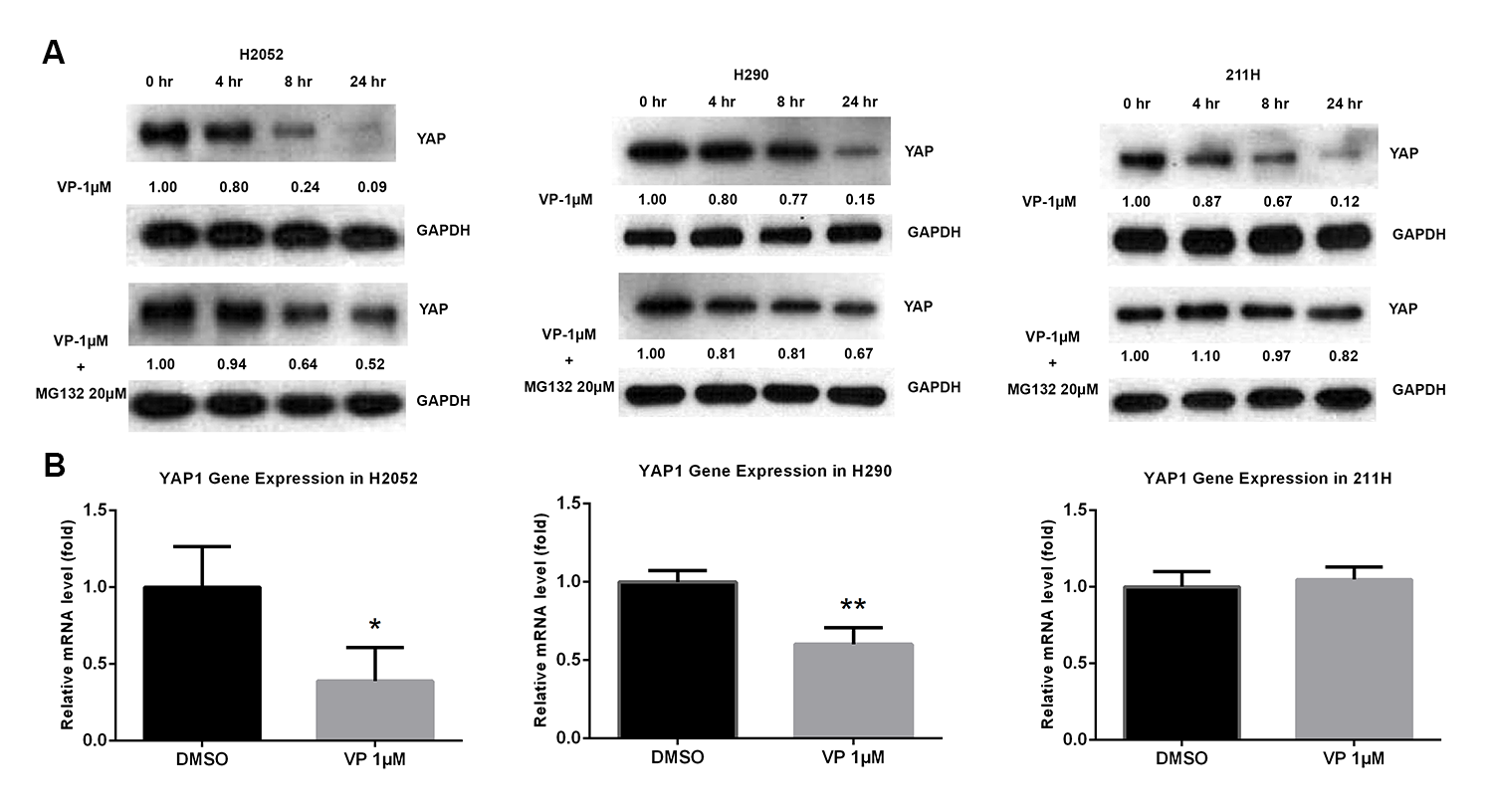

Supplement: Supplementary file 1 — Fig. S1 Analysis of YAP protein level and mRNA level with 1 μM verteporfin treatment alone or 1 μM verteporfin with 20 μM MG132. [file JCMM-21-2663-s001.tif]

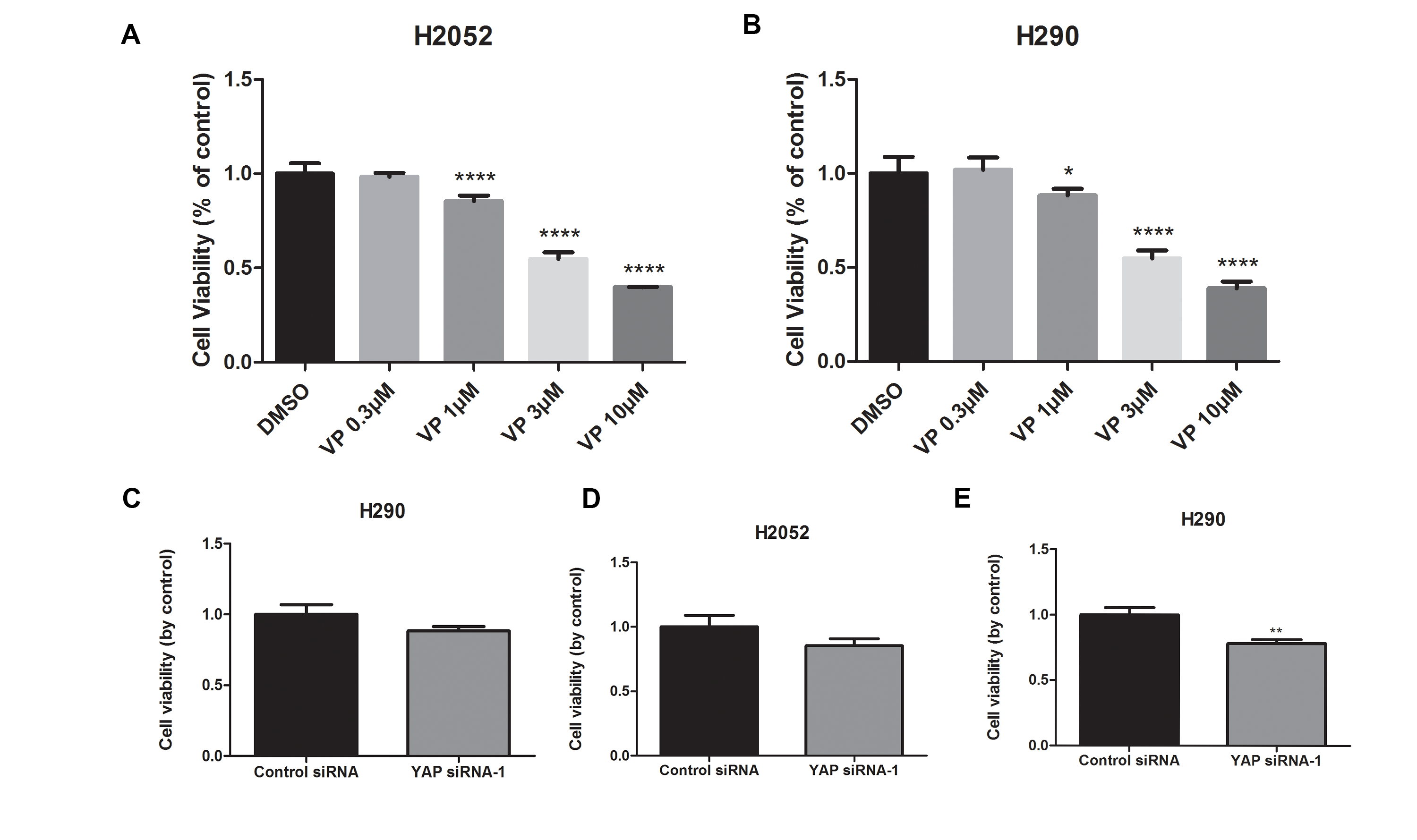

Supplement: Supplementary file 2 — Fig. S2 Cell viability of H2052 (A) and H290 (B) treated with verteporfin for 24 hrs. (*P < 0.05, ****P < 0.0001, one‐way anova and Scheffe multiple comparisons). H290 and H2052 cells were treated with control siRNA or YAP siRNA‐1 for 24 hrs. Equal cell numbers (5000 cells in 500 μl) of H290 and H2052 were seeded in 96‐well plates. After 20 hrs, cell viability of H290 (C) and H2052 (D) were analysed. After 7 days, cell viability of H290 (F) was analysed. [file JCMM-21-2663-s002.tif]

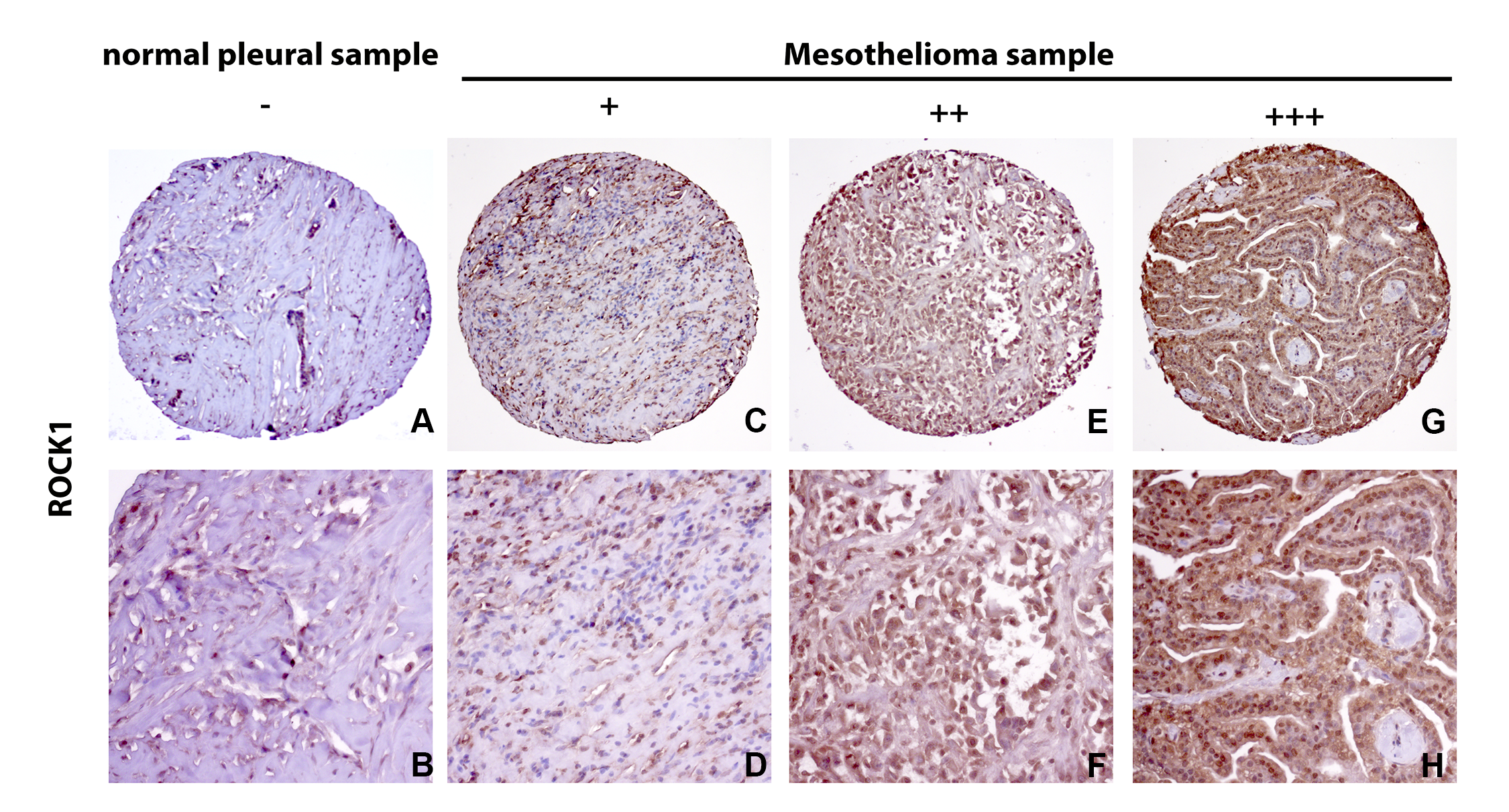

Supplement: Supplementary file 3 — Fig. S3 Immunohistochemistry of ROCK1 staining in mesothelioma and normal pleura samples. [file JCMM-21-2663-s003.tif]
